# Personal Health Tracking: A Paradigm Shift in the Self-Care Models in Nursing

**DOI:** 10.2196/50991

**Published:** 2023-09-20

**Authors:** Soyoung Choi

**Affiliations:** 1 Department of Kinesiology and Community Health University of Illinois at Urbana-Champaign Urbana, IL United States

**Keywords:** personal health data, personal informatics, self-care, self-tracking, mobile health technology, human-technology, human-computer, human computer interaction, health tracking, framework, frameworks, model, models, mHealth, mobile health, informatics

## Abstract

The rapidly evolving digital health landscape necessitates updates to existing self-care models in nursing. This viewpoint paper revisits and evaluates prevalent models, recognizing their comprehensive exploration of self-care concepts while also identifying a gap in the incorporation of personal informatics. It underscores the missing link of human-technology interplay, an essential aspect in understanding self-care practices within digital generations. The author delineates the role of personal health tracking in self-care and the achievement of desired health outcomes. Based on these insights, the author proposes a refined, digitized self-care model that incorporates mobile health (mHealth) technologies and self-tracking behaviors. The paper concludes by advocating the application of this model for future mHealth nursing interventions, providing a framework for facilitating patient self-care and improving health and well-being in the era of digital health.

## Introduction

Self-care refers to the personal practices adopted to maintain and enhance health [1], whereas self-management not only covers self-care but also delves into facets like emotional and role management in chronic illness [2]. In nursing, especially concerning chronic conditions, both concepts are crucial, allowing patients to actively engage in their health care and thus improving health outcomes [1]. Dorothea Orem’s self-care model [3] has ushered in a significant shift in health care dynamics, emphasizing individual empowerment in managing health and well-being. Rooted in the belief that individuals inherently possess the capacity for self-care, the model contends that they can make informed decisions to improve their own health. Central to this model is the principle of self-care, which sees individuals not merely as recipients of care but as active participants in their own health maintenance. Within this paradigm, the role of nurses evolves from care providers to facilitators, educators, and advocates, empowering patients to cultivate the skills and knowledge necessary for effective self-care [4].

Barbara Riegel’s self-care model [5] is a modern theoretical framework that accentuates the importance of self-care in managing chronic illnesses, specifically in the context of heart failure. Offering a holistic view of self-care and its implications on managing long-term conditions and optimizing health outcomes, this model delineates 3 interconnected dimensions of self-care: maintenance, monitoring, and management [5]. These elements collectively foster self-care practices for those grappling with chronic illnesses. Contrary to perceiving self-care as an exclusively individual endeavor, Riegel’s model acknowledges the substantial influence of socioenvironmental factors and underscores the crucial role health care providers and support systems play in facilitating productive self-care behaviors. By empowering patients and boosting their confidence in self-care, nurses can stimulate active involvement in health management and engender improved health outcomes.

The patient-centered approach embodied in current self-care models in nursing has gained significant recognition for promoting individual autonomy and self-determination [6]. In recent years, the advent of mobile health (mHealth) technology and the quantified self movement [7] has expanded the potential of self-care models, enabling individuals to manage and monitor their health using mHealth devices and applications. Consequently, the integration of mHealth technology has contributed to a substantial transformation within the nursing profession’s self-care model. This phenomenon has redefined the methods by which individuals manage their health, thereby empowering them to take proactive roles in their health and well-being [8-10]. With the aid of mHealth applications, wearable devices, and other digital tools, patients and caregivers now possess the ability to access real-time health data, personalized health information, and track their progress toward health goals. This new trend has not only bolstered patient engagement and satisfaction but has also unveiled new prospects for health care providers to deliver personalized care [11].

This paper delves into the interplay between mHealth technology and personal health tracking within the self-care models in nursing, introducing the newly emerged digitized self-care model. The author explores the potential advantages and barriers of integrating these advancements into nursing practice, paying particular attention to their effects on patient outcomes, health care accessibility, and the dynamics of the health care provider-patient relationship. By understanding the paradigm shift brought about by these technological advancements, nurses can adeptly integrate and leverage mHealth technology, thus aiding individuals in their pursuit of optimal health and well-being, informed by insights from personal health data in their everyday lives.

## Background

The advent of the quantified self movement [7] has paved the way for a novel paradigm, wherein individuals use mHealth technologies to track and scrutinize their daily activities, thereby attaining an enriched understanding of their comprehensive well-being. This movement, embodied in quantified self technologies (QST), hinges on the systematic collection and analysis of personal data. Buchanan and Lockton [12] pinpoint three interconnected attributes intrinsic to QST: (1) feedback, which concerns the provision of information; (2) connectivity, which revolves around how individuals leverage QST to monitor, share, and elevate their health; and (3) intervention, which involves psychological determinants such as user motivation and habitual behaviors. This trend denotes an inclination to quantify everyday activities such as eating, sleeping, and exercising, assigning tangible numerical data to them [13]. By incorporating elements such as activity trackers and digital biomarker collection into their lifestyles, quantified self proponents perceive these as invaluable instruments that yield concrete data pertaining to myriad facets of their daily routines [14].

Li’s stage-based model [15] is a renowned framework in personal informatics, delineating 5 psychological stages integral to engaging with digital self-tracking. The initial phase, termed “preparation,” encompasses individuals’ motivations to accumulate personal data, their decision-making process in choosing pertinent data, and their strategies for its documentation. Subsequently, in the “collection” phase, individuals amass self-related data. “Integration,” the ensuing phase, prepares, amalgamates, or modifies the collected data to ease reflection. In the final “action” stage, individuals make informed decisions based on their improved self-awareness. Li et al [15] underscore a noteworthy observation that individuals frequently concentrate on a specific phase, such as logging the number of steps taken or hours slept, while neglecting the comprehensive process and anticipated health outcomes of self-tracking, thereby stressing the need for professional guidance and continued support. Additionally, Li et al [15] highlight the potential role of computers in streamlining self-tracking citing progress in sensor technologies, the ubiquity of internet-enabled information, and the advent of user-friendly systems and interfaces. Subsequently, Epstein et al [16] proposed an updated version of Li’s stage-based model, termed the “lived informatics model.” This refreshed framework introduces additional stages—“deciding,” “selecting,” “tracking and acting,” and “lapsing.” The integration of these phases offers an expansive insight into how individuals use self-tracking tools, uncovers motivational strategies, and complements the original stage-based model in tracing behavioral changes.

The management of personal health data plays a crucial role in the broader health care landscape. As delineated by the “illness work and personal information management” framework [17], patients undertake a range of complex self-care activities. These tasks span several responsibilities including, but not limited to, adhering to medication regimens, refilling prescriptions, shopping for groceries and preparing meals, exercising, or undergoing physical therapy, navigating medical challenges, identifying health care providers, scheduling medical appointments, and maintaining medical records. Consequently, patients contending with multiple chronic conditions often encounter additional challenges and feelings of being overwhelmed when tasked with managing their health-related information. Early on, Corbin and Strauss [18] emphasized the importance of “articulation work” within the scope of “illness work,” particularly stressing its role in the use of technologies.

Prior research illustrates several advantages offered by using mHealth technologies to monitor daily activities, especially concerning the automatic assembly and aggregation of data [19]. For instance, the variety of mHealth applications available assists both patients and health care providers in selecting the application best suited for their needs. In terms of function, there are diagnostic applications that assist in identifying illnesses, health behavior change applications aimed at fostering healthier habits, symptom checkers that guide users based on their current symptoms, and specialized apps for managing chronic conditions such as diabetes or asthma. Depending on the characteristics of health data, passive applications automatically collect data using sensors, while active applications require individuals to manually input data, like diary entries. Based on the responsibilities and central roles in self-care, the roles of patients in using mHealth technologies can vary [1]. For example, when reshaping healthy lifestyles, patients bear greater responsibilities. Conversely, in acute care and serious illnesses, the responsibilities predominantly fall on health care professionals [1]. The Good Practice Guidelines on Health Apps and Smart Devices [20] offer recommendations for the design, deployment, and adoption of mHealth technologies aiming to enhance their use in health care environments and improve health outcomes.

## A Digitized Self-Care Model

### Overview

Existing self-care models need some updates to adequately address the influence of personal informatics and personal health tracking in the context of nursing. For instance, while the middle-range theory of self-care for chronic illness [4] comprehensively addresses the self-care process in patients with chronic conditions, it does not encompass the recently developed field of personal health informatics [21]. The vital interaction between personal health tracking and self-care is notably missing element, hindering the understanding of factors influencing self-care in the digital age. To bridge this gap, this paper scrutinizes published papers on patients’ health-tracking behaviors to comprehend how personal informatics is reflected in their self-care practices and desired health outcomes. In light of these findings, the author proposes a digitized self-care model and recommends its application for future mHealth-based nursing interventions.

### Personal Health Tracking

In this paper, the term “personal health tracking” is favored over “self-tracking” by the author. This preference is intended to more accurately represent self-monitoring behaviors within the scope of self-care, specifically using mHealth technologies and quantified health metrics. Essentially, in the context of this study, “personal health tracking” denotes the practice of individuals leveraging mHealth technologies to collect personal health data with the intention of addressing abnormal health issues or enhancing their overall health.

Personal health data cover a range of health-related metrics that individuals can autonomously monitor in their daily lives, such as heart rate, body temperature, blood oxygen saturation, sleep patterns, physical activity or exercise, food consumption, and mood states [22]. The practice of observing and collecting these data using digital technologies and tools is typically termed “self-tracking” [23]. Historically, patients relied on traditional pen-and-paper methods to document their symptoms and perceived status. The modern approach, however, involves wearing devices that capture diverse health metrics, considerably alleviating the workload associated with data collection. For example, individuals can now wear fitness trackers that automatically record heart rate, sleep patterns, step count, and daily calorie burn. These devices collect data in real time, eliminating the need for manual logging. Beyond capturing health metrics and behaviors, there is a growing emphasis on patient-reported outcomes—direct reports from patients regarding their health status [24]. Health care providers are increasingly valuing these patient-reported outcomes, recognizing that the patient’s voice is vital for patient-centered care [24]. While a segment of the older population continues to use manual methods [25], this unobtrusive capture of health data mitigates the traditional tracking burden, offering users a more convenient and accurate means to oversee their health and behaviors.

**Figure 1:**
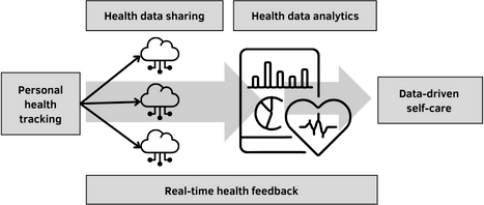
The digitized self-care model.

The digitized self-care model (Figure 1) highlights the intersection between the concept of self-care monitoring [5] and personal health tracking. As defined in the self-care model, self-care monitoring involves the practice of self-observation for alterations in signs, symptoms, or regular bodily monitoring [4]. They emphasize that self-care monitoring incorporates vital elements such as detectable health conditions, dependable methods for identifying bodily changes, and the capacity for appropriate response [5]. While individuals may detect physical alterations through various means, quantified measures are deemed reliable health data for health care providers in evaluating and responding to these changes. Nonetheless, the lack of contextual information in personal health tracking has drawn criticism from health scientists [26]. Therefore, the synthesis of contextual information and health data is crucial in comprehending individuals’ health conditions and related behaviors [27].

In the digitized self-care model, personal health tracking can be conducted either short-term or long-term, depending on the person’s intent and determination. “Selective personal health tracking” refers to the temporary collection of personal health data to reflect diagnosis and management. Meanwhile, “prolonged personal health tracking” denotes the ongoing monitoring of physical changes, health indicators, and disease conditions. Selective personal health tracking, which involves a relatively small volume of personal health data, primarily monitors current illness progress, gauges treatment effectiveness, and aims to achieve desired health outcomes in a brief period. This approach is less about discerning meaningful health-related patterns from accumulated health data for predictive purposes. In contrast, prolonged personal health tracking does not focus on attaining immediate health status changes, but primarily aims to continuously monitor physical changes for health promotion and to maintain health status within a distinct adjunct to care. Therefore, the duration of personal health tracking is determined by individuals based on their health and health care needs.

### eHealth Literacy, Technology Literacy, and Data Literacy

eHealth literacy, technology literacy, and data literacy are important skills in the digital age. eHealth literacy centers on the capability to seek, retrieve, comprehend, and evaluate health information from electronic sources [28]. Technology literacy pertains to proficiency in interacting with technology [29], and data literacy encompasses the aptitude to comprehend, analyze, and interpret data [30]. These competencies are crucial for individuals to make informed decisions, especially in the health care sector. The digitized self-care model starts with the premise that patients have the basic literacy expected in the digital age, coupled with individuals’ motivation necessary for basic self-care practices. This pertains not just to a fundamental comprehension of one’s technology usage and health data interpretation, but also its application in subsequent self-care behaviors. The extent of an individual’s education, their inclination toward health care knowledge, and their comprehension of it serve as crucial components for successful personal health data-driven self-care. Furthermore, it is important to assess individuals’ access to technologies and financial constraints, as these factors can pose challenges to their engagement with mHealth-based self-care.

In clinical practice, the adoption of mHealth technologies for personal health tracking is influenced by factors such as health care needs, attitudes toward mHealth technology, as well as the literacy levels of individuals [31]. Health care providers should be aware of the patient’s capabilities and preferences to recommend appropriate mHealth tools. Assessing the patient’s context and clinical care information is essential in determining the most effective approach to their self-care. In the realm of digital health, nurses will have a critical role in evaluating patients’ information and selecting the most suitable mHealth tools to support their self-care activities. Additionally, it is crucial to educate patients who may have difficulties in understanding their health data, as this is vital for successful digitized self-care. Furthermore, efforts to enhance patients’ self-efficacy in acquiring new technological skills and interpreting health data should be an integral part of this process.

As we embrace the evolution of mHealth technology in health care, we also risk widening health disparities across diverse populations. This is partly due to the fact that the intellectual gap among individuals capable of leveraging these technologies is not being adequately addressed. It is paramount that when introducing mHealth technologies to patients, their foundational literacy skills are evaluated to ascertain their capacity to use these technologies in self-care practice. Only when individuals can independently use mHealth technologies and make sense of the personal health data collected, can they fully comprehend their personalized care plans and take responsibility for their self-care. Moving forward, incorporating assessments and education regarding patients’ eHealth literacy, technology literacy, and data literacy will become an essential component of clinical practice.

### Personal Health Data-Driven Self-Care

The essence of the digitized self-care model involves collecting and analyzing personal health data over varying periods, thereby enabling constructive self-care. Self-care maintenance, self-care monitoring, and self-care management [5] are founded on the quantified health metrics derived from the collection and analysis of personal health data. While intuition and self-care insights from past experiences can influence self-care planning and practice, it is the interpretation of personal health data that has a more profound impact. Health data collected during an individual’s daily routine are shared with health care providers and caregivers. With the appropriate patient consent, personal health data stored in the cloud can be accessed by third parties. This facilitates person-centered care through a process commonly known as health data sharing [32]. The accumulated personal health data can be harnessed to deliver predictive care plans to patients and caregivers. This is made possible by using data mining techniques that are powered by big data.

Thanks to real-time access and analysis capabilities in mHealth systems, patients can receive immediate personalized feedback and medical alerts [33]. Such real-time data analytics technology is gaining momentum in the health care sector, empowering patients with proactive self-care tools. Personal health data–driven self-care increases the likelihood of achieving expected health outcomes, and prediction of health conditions may aid in the early detection and prevention of diseases [34]. Consequently, self-care effectiveness and efficiency can be maximized by analyzing personal health data, rather than relying solely on the existing medical knowledge and professional experience of health care providers. This real-time health feedback feature, which encompasses health data sharing and health data analytics, shares characteristics commonly found in consumer health informatics [35]. Consumer health informatics integrates remote monitoring systems, patient medical records, decision support systems, and web-based health communities to facilitate individual self-care [36]. This is realized through the amalgamation of daily routines, concurrently focusing on patients, family members, associated daily activities, and the surrounding context. In contrast to the consumer health informatics applications, the digitized self-care model places more emphasis on the ability of persons to collect their own health information and use it as a means to improve their health and well-being.

As previously noted, the digitized self-care model’s fundamental objective is to guide researchers to incorporate the concept of personal health tracking in the design of mHealth-based self-care interventions. Previous mHealth studies have not primarily focused on patient access to, and comprehension of, personal health data. Instead, the collected health data have predominantly been used by health care providers and researchers for evaluating patients’ health status or measuring intervention effectiveness. In the majority of prior studies, patients have not been active governors of their health data. This novel model, however, underscores the significance of patients becoming active participants in mHealth technologies. It advocates for patients to voluntarily monitor their health data, stay attuned to changes in their bodies, and take an active role in the self-care process.

**Figure 2:**
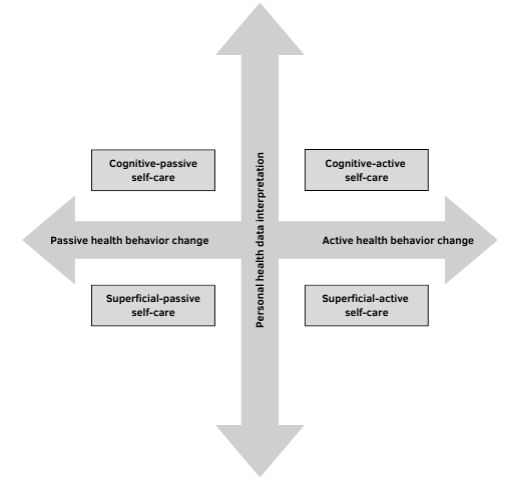
Types of data-driven self-care.

The health data-driven self-care can be classified into four primary categories based on the extent of an individual’s engagement in interpreting health data and implementing health behavioral changes (Figure 2): (1) cognitive-active self-care, where an individual grasps the underlying meaning of their health data and translates it into subsequent health behavior changes (eg, patients modifying their dietary intake in response to blood glucose readings); (2) superficial-active self-care, which involves a superficial understanding of one’s health data, followed by implementing health behavior changes as mainly guided by health care feedback (eg, patients who respond to messages from an app recommending 30 minutes of walking); (3) cognitive-passive self-care, in which an individual deeply comprehends the significance of their health data but does not initiate any health behavior change (eg, patients understanding a consistent weight gain trend from the health chart but do not show any behavioral modifications); and (4) superficial-passive self-care, where an individual merely glosses over their health data without instigating any health behavior change (eg, patients who use wearables without demonstrating enhanced health literacy or behavioral modifications).

As per the self-experimentation framework in personalized health [36], individuals who are cognitive and active go beyond simple self-tracking. They endeavor to comprehend their present or potential health issues and possibly take effective measures to optimize health outcomes. These individuals generate hypotheses to address their health-related queries and use their own bodies to ascertain the causes of health problems. This advanced level of self-care requires individuals to possess a superior understanding of medical knowledge and logical thinking skills to conduct “self-experiments.” In fact, individuals capable of practicing this advanced level of self-care are rare. Laypersons are often confronted with misleading medical information and struggle with assessing and navigating the surfeit of medical misconception [37]. As a result, patient’s self-care activities must be supplemented with the guidance of health care professionals, assisting them in correctly interpreting their health data and devise actions to enhance their health and well-being. It is crucial to acknowledge that misuse of data and information can promote detrimental health behaviors and result in unforeseen negative health outcomes. Additional educational opportunities and counseling should be provided to patients and caregivers to ensure that digitized self-care outside of the health care setting—be it at home or in the community, in realms beyond the influence of health care professionals—is executed appropriately.

## Clinical and Research Implications

Without a doubt, not all individuals possess the ability to engage in digitized self-care. Currently, various marginalized groups in our society, including the older populations, people with disabilities, and low-income individuals face challenges in using new digital technologies and frequently updated software. Hindered by lower education levels and financial constraints, they are often unable to adopt innovative technologies. Health care systems and health insurance plans must persist in providing financial support and assistance to these marginalized groups, thus enabling greater access to innovative technologies that can facilitate self-care and health improvement. Moreover, encouraging patients to monitor their personal health data can be challenging for various reasons. A primary obstacle can be the skepticism patients might have toward mHealth technologies’ accuracy and reliability for tracking health metrics and behaviors. This skepticism can demotivate them from collecting and sharing their health data with health care professionals. Further research is required to determine how to encourage patients to use mHealth technologies for self-care. Nonetheless, all individuals in the digital age should have equal opportunities to use cutting-edge technologies, such as mHealth, to enhance their quality of life.

The adoption of mHealth technology for patients’ self-care in clinical and community settings can pose several challenges for nurses. These challenges include infrastructure constrains, technological disparities, privacy issues, and patient-related factors. Significantly, there is scant research regarding nurses’ comprehension of mHealth technologies and their proficiency in effectively using these tools within self-care routines. Within the domain of nursing education, it is imperative to emphasize the importance of systematic training to address the technological divide, equipping nurses with the confidence to adeptly use these tools in patient care. Additionally, as mHealth platforms are integrated into health care, the transmission of health data introduces potential ethical concerns. It remains crucial for nursing educators to underscore the importance of maintaining data privacy and confidentiality to secure patient trust and align with professional ethical standards.

Collaborating with diverse experts is crucial to address the needs of data security, data governance, and interoperability in the adoption of mHealth technologies in patient care. Data security is pivotal in mHealth technologies due to the handling of sensitive patient information. Collaboration with cybersecurity and data privacy experts is essential for preparing strong protective measures. Clear protocols for data handling are essential for preserving its integrity, privacy, and confidentiality. Experts in data governance and ethics can guide on aspects like ownership, consent, and compliance with regulations, including the Health Insurance Portability and Accountability Act. Finally, interoperability can be a challenge in mHealth due to varying data formats across systems and devices. Collaborating with health informatics experts can help create standardized data exchange protocols and the designs of systems that allow data sharing across diverse health care platforms.

## Conclusions

In conclusion, amidst the deluge of mHealth technologies available to the public, this paper presents a digitized self-care model for the contemporary era. This updated self-care model merges the established self-care model with the concept of personal health tracking, with an aim to enhance self-care practices for both patients and health care providers. This model eliminates extraneous research concepts, providing researchers or practitioners with flexibility when crafting nursing interventions. It can further be used by incorporating significant external factors affecting self-care as valued by health care scientists. The core of personalized care is diminished when patients forgo their agency. However, by empowering patients to actively collect, understand, and use their health data to modify problematic self-care activities, they can maintain optimal health outcomes. As we progress in the digital age, marked by unpredictable advancements in artificial intelligence and internet of things, existing nursing models will need frequent updates to remain relevant and effective.

## Abbreviations

mHealth: mobile health
QST: quantified self technologies
